# Midwives’ work-related fear and anxiety and its impact on their wellbeing and performance. A qualitative study of perceived anxiety in community midwives

**DOI:** 10.18332/ejm/172574

**Published:** 2023-11-20

**Authors:** Davita H. van den Heuvel, Liesbeth E. Kool, Tamar L. Nelson, Esther I. Feijen - de Jong

**Affiliations:** 1 Academy Midwifery Amsterdam and Groningen, Groningen, The Netherlands; 2 University of Groningen, University Medical Center Groningen, Department of Primary and Long-term Care UMCG, Groningen, The Netherlands; 3 Amsterdam UMC, Vrije Universiteit Amsterdam, Department of Midwifery Science, Amsterdam, The Netherlands

**Keywords:** midwives, fear, anxiety, clinical decision-making, risk assessment

## Abstract

**INTRODUCTION:**

Working with acute situations is usually part of midwifery practice. In the Netherlands the community midwives work in a context where they are mostly the sole decision-makers and policymakers and often do not have the support of a multidisciplinary team during a birth. How Dutch community midwives maintain their emotional hygiene is not known. This study aims to explore how Dutch midwives perceive fear and its influence on their performance.

**METHODS:**

This is a qualitative study with semi-structured interviews of 19 Dutch community midwives between October 2018 and January 2019.

**RESULTS:**

Four themes were identified: 1) midwives’ perceptions of fear and anxiety, 2) how years of experience affect fear and anxiety, 3) influence of the work content; and 4) implications for performance. Midwives perceived fear in acute situations where maternal and/or fetal complications were imminent. Participants perceived anxiety either as helpful or a hindrance. Awareness of these feelings helps them to regulate whether or not to give in to these feelings.

**CONCLUSIONS:**

Our findings suggest similar perspectives on fear in Dutch community midwives compared to previous outcomes. In the Netherlands, midwives seem reluctant to talk about fear and anxiety in the profession. The awareness of these emotions occurring while working is essential for the wellbeing of midwives, as well as the importance of knowing how to act on fear and anxiety.

## INTRODUCTION

Over the past decades, societal trends and international comparisons of maternity care outcomes have had an impact on midwifery care and midwives in the Netherlands^1,2^. The focus of community midwifery in the Netherlands has shifted from a physiological perspective to mitigating obstetric risks and reducing perinatal mortality^3^. Furthermore, this shift may have contributed to a decline in home births and an upward trend in interventions during birth^3^. Moreover, the emphasis on risk reduction has also influenced midwives’ perception of pregnancy and childbirth as inherently risky events at the expense of confidence in the physiological processes^2,4^.

The shift in society and healthcare towards reducing childbirth risk might affect the experience of fear or anxiety among midwives^4^. Studies on fear among midwives show that high levels of fear are correlated to reduced confidence in midwives in being able to support birthing parents^3,5^. Fear is hereby defined as a response to a specific danger^6^. Studies show that fear in midwives manifests itself in several ways: 1) fear of perinatal mortality^7^, 2) fear of missing information and causing harm to pregnant women^7-9^, 3) fear of obstetric emergencies and maternal mortality^7^; and 4) fear of blame for incidents, and legal ramifications^7,10,11^. Fear seems to have a direct effect on the performance of physicians, for instance^12^, who, as a result, have a higher rate of medical errors and exhibit less empathy.

In the Netherlands, research suggests that midwives’ perceptions of anxiety, along with increased protocols and guidelines, are the most important contributors to increased referrals of low-risk pregnant women to hospital^3^. Whilst fear is a reaction to a specific danger, anxiety is experienced as a more general, aimless, and future-oriented feeling, which results in a positive or negative influence^6^. A review indicates that community midwives exhibited higher anxiety levels than hospital-based midwives^13^. The autonomous working method of community midwives triggers these feelings of anxiety and causes midwives to act defensively^13^. Furthermore, negative individual birth experiences could affect midwives’ risk perception, which can cause midwives to act more cautiously^14^.

Little is known about Dutch midwives’ perceptions of fear and anxiety in the community and its impact on their performance. Although previous studies explored fear in midwifery and the relationship between fear and decision-making^9^, the predominantly solo work of Dutch community midwifery might influence the experience of fear and anxiety in practice. There appears to be an absence of research in this area. The aim of this study, therefore, is to explore work-related fear and anxiety among Dutch community midwives using the following research question: ‘How do Dutch community midwives experience work-related fear and anxiety, and what is its perceived influence on them?’.

Research and attention to this sensitive subject can contribute to efficient management of fear and anxiety in midwifery and the impact on the quality-of-care midwives provide for pregnant women and their families.

### Midwifery in the Netherlands

In the Netherlands, maternity care is divided into primary and secondary midwifery care^15^. Community-based midwives work in primary care for women with low-risk pregnancies. Community-based midwives can work either self-employed, employed or as a locum midwife^16^.

Community-based midwives assess women and allocate them into evidence-based risk categories^1^. Women who belong to the group of low-risk pregnancies remain in primary care and they can choose whether to give birth at home or in hospital with support of a community-based midwife. Hospital-based midwives provide care to women with mid- and high-risk pregnancies under supervision of an obstetrician in the hospital^15^.

The Netherlands is a densely populated country with almost 4000 midwives working in an area of approximately 41500 km^2^. It is estimated that about 25% of this total group work in hospitals^17^. There are 81 hospitals in the Netherlands^18^. The applied standard of starting treatment in a hospital is as follows: 24/7 must be started within an acceptable time (currently based on a 45-minute standard, where the ambulance response time is 15 minutes)^19^.

## METHODS

**Figure 1:**
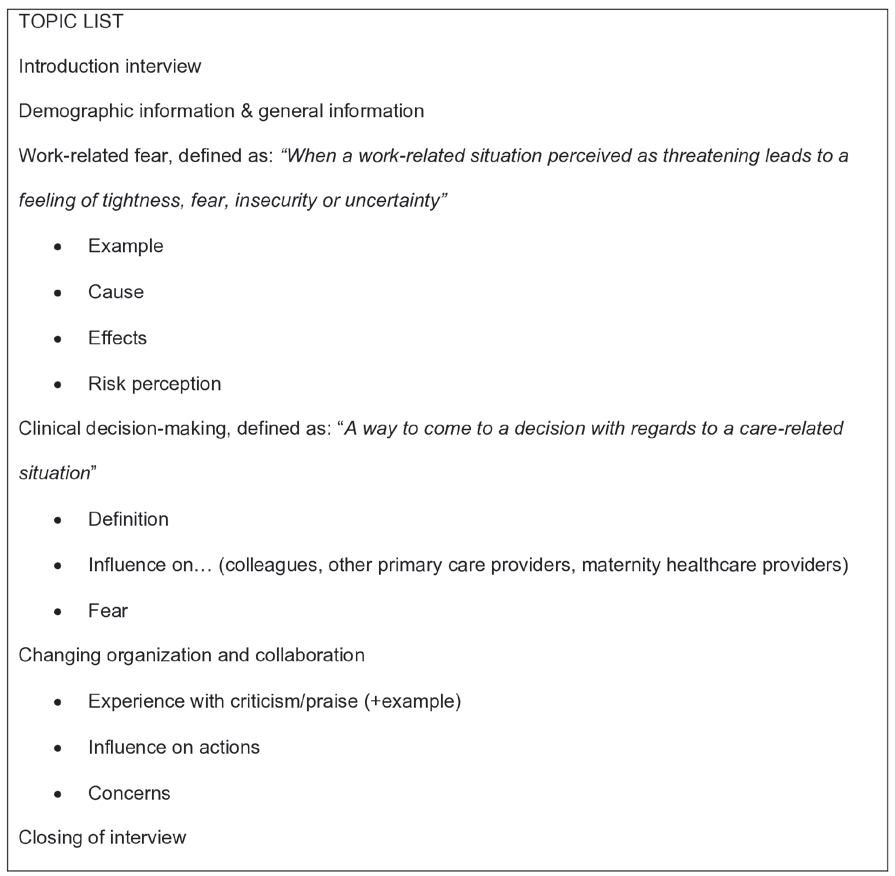
Topic list of fear and anxiety among Dutch community midwives

We conducted exploratory qualitative research using interviews guided by a list of topic areas. The interviews were semi-structured as we used a list of topics (Figure 1) based on a literature review. We began each interview with open-ended questions about their fear and anxiety. With this list of topics, we were able to use the topics as prompts for participants to reflect on specific aspects of fear and anxiety^20^. The study was reported using the SRQR checklist^21^.

During the data collection, the topic list changed. At first, the interviews were performed with only open questions about fear. The interviewers noticed that fear was not a static feeling, but can be experienced in both fear and anxiety. Therefore, we added topics to the topic list.

The interviews were conducted by twelve midwifery students (students of an honors program) who were trained in qualitative interview techniques beforehand to ensure consistency in the interviews. The students were supervised by two researchers: a social scientist and a medical doctor. A higher level of consistency was achieved because the two supervisors trained the students and prepared the topic list with the students.

Participating midwives were recruited from midwifery practices nationwide through convenience sampling by using the students’ networks. Snowball sampling was also used, with midwives nominating other midwives for the study. To obtain a good variety in our sample, we also selected by age, number of years of experience, number of colleagues, and urban or rural areas. Twenty-six midwives were asked to participate in the study. Six declined due to logistical reasons and time restraints. Twenty midwives were interviewed between October 2018 and January 2019. Sixteen midwives were interviewed individually. For logistical reasons, two interviews were conducted in pairs, as requested by these participants.

All interviews were audio recorded and transcribed. Prior to analysis, a member check was conducted by sending the transcript to the midwives who agreed to read it. One participant withdrew the interview due to concerns about privacy and the sensitive nature of the information provided. Other participants agreed to their transcripts.

Thematic content analysis was used for this study^20^. First, two transcripts were individually coded by groups of four students, and the remainder of the interviews in pairs. Any discrepancies in coding were discussed within the whole team until a consensus was reached. The analysis followed an inductive bottom-up approach according to the stages of content analysis.

The final coding tree and themes were agreed upon by the students and their supervisors. MAXQDA 2020 was used to analyze the transcripts. In this entire process, each step was supervised and assessed by the accompanying supervising researchers.

### Ethical considerations

No ethical approval is required for this type of research in the Netherlands, as no patients are involved. Only healthcare professionals participated in this study^22^. However, we had to protect the anonymity of the professionals involved and also meet the requirements for data security. The Medical Ethics Board of the University Medical Centre Groningen has declared that this study meets the necessary requirements (METc2023/440).

Prior to the start of the interviews, each midwife was asked to sign a participation agreement of informed consent, and to complete questions to gain basic demographic information. Midwives could withdraw from the study if they wished to do so. The interviews were transcribed anonymously; no personal identifiers were included in the transcriptions in order to comply with confidentiality requirements. During the entire study, confidential information was stored on a secure server of the University Medical Center Groningen or destroyed immediately after analysis. Only the principal investigator (LK) has access to the personal characteristics and can link names to participant number.

## RESULTS

Table 1 shows the background characteristics of the participants. The participants vary in age, number of years of work experience, and working areas (urban/rural). There were more midwives aged 40–49 years and more self-employed midwives among the participants compared to the population of midwives in the Netherlands^23^. The percentages of participants who worked in duo and group practices were almost equal.

**Table 1:** Background characteristics of study participant midwives and of the population of Dutch midwives

*Characteristics*	*Participants (N=19)*	Dutch midwives (N=3638)*
	*n (%)*	*n (%)*
**Age** (years)		
<30	5 (26)	941 (26)
30–39	3 (16)	1264 (35)
40–49	8 (42)	741 (20)
≥50	3 (16)	692 (19)
**Sex**		
Female	19 (100)	3157 (98)
Male	-	64 (2)
**Experience years in midwifery**		
<5	5 (26)	
5–10	4 (21)	
>10	10 (53)	
**Type of practice**		
Solo		(4)^a^
Duo	2 (11)	(11)^a^
Group	16 (84)	(85)^a^
Caseload	1 (5)	
**Urbanization level**		
Urban	9 (47.4)	
Rural	9 (47.4)	
Urban and rural	1 (5.3)	
**Employment status**		
Employed	1 (5)	1180 (36.8)
Self-employed	18 (95)	1444 (45.0)
Locum	-	583 (18.2)

* Kenens et al.^23^ (2020) Statistics from the Midwifery Register.

a Only percentages were available.

**Figure 2:**
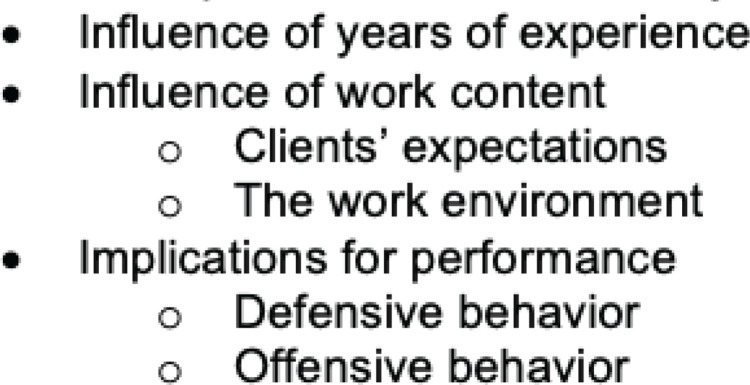
Code tree on the study of Dutch community midwives’ perceptions of fear and anxiety (N=19)

Data saturation at the code level was reached after fourteen interviews. The remaining three interviews were then coded to ensure that no new information emerged. After analyzing all the transcripts, we constructed four main themes: perceptions of fear and anxiety, influence of years of experience, influence of the work content, and implications for performance (Figure 2).

In general, participants perceived fear and anxiety at work. Some participants acknowledged the feelings of fear or anxiety, while others seemed to recognize these feelings only in fellow midwives. During the interviews, it appeared that talking about fear and anxiety in midwifery for participants was not common practice.

### Perceptions of fear and anxiety

Participants perceived fear and anxiety as two different feelings. Firstly, they described fear as an emotion that occurred in situations with obstetric emergencies, in particular with a negative outcome involving fetal or maternal complications. They felt an awareness and responsibility that they have to resolve this situation on their own while working alone as a midwife in the community:

*‘And eventually the child comes, well, at that time I am afraid in a way like, oh dear, can't get him out, but I don't think I fully realize at that moment. It's more after that time, I think.’* (P13)

Secondly, participants described feelings of anxiety as: 1) a healthy tension that helped them respond to a situation, also referred to as being extra alert to identify potential risks, and 2) increasing anxiety due to the risk. In participants’ perceptions, the increasing anxiety was attributed to a shift in maternity care and in society. They mentioned that they had to adjust to more risk-avoidance behavior. For instance, in midwifery practice, this shift has led to working according to strict protocols. Furthermore, participants wondered whether feelings of anxiety were positive or negative as a guide for their work in practice:

*‘Should I do anything with this, should I not. Well, I wouldn't call it fear, it's more like identifying potential risks, but you do notice that your adrenaline goes up, and your heart rate goes up, and you're super alert. … And when I hear people say they are not afraid at all or know no fear, I think, “how alert are you then?”.’* (P9)

### Influence of years of experience

Participants perceived years of experience as an important factor in the perception of fear and anxiety. It could increase or reduce fear and anxiety. Participants stated that the number of acute situations increased their doubts about their ability to handle an acute situation. In addition, they mentioned the unpredictability in these situations as triggers for fear and, in the longer term, for anxiety. A participant talked about the effect of a previous fearful situation on her subsequent actions:

*‘Well, I'm not really thinking anything because then you just have to wait for a contraction, obviously, and that's ok, and that you then try to get the child going. Maybe you do that more consciously then. Consciously really move that shoulder more towards the sacrum and then towards the symphysis pubis. ... Yes, er, I do think when I look back … that you are always more alert, but that may well be the same if I have just had someone with heavy bleeding. That you are alert to that.’* (P5)

On the other hand, years of work experience could also lead to decreasing anxiety, as described by participants. They perceived that they experienced more anxiety when they were newly qualified compared to their more experienced years. Other participants mentioned that they perceived more anxiety when returning to practice after a period of leave:

*‘Well, what I see in myself is when I was just starting out, I found it much tenser than I find it now. Then I had, um, more trouble blocking that fear, um, or reducing it. And now I'm much better at that.’* (P3)

### Influence of work content

*Clients’ expectations*

Participants perceived that sometimes the communication with pregnant women could contribute to feelings of anxiety. A relationship without a solid trust, and poor communication between the midwife and the parents could increase participants’ anxiety. Participants mentioned other issues related to pregnant parents’ expectations. Some clients had expectations of giving birth that did not meet the professional guidelines. For instance, the wish of parents to give birth at home while they were referred to secondary care due to obstetric complications. These wishes resulted in increased anxiety for some midwives:

*‘Occasionally, clients approach me with specific concerns about certain aspects of their obstetric care [expectations not in line with professional guidelines]. In response, I engage in a dialogue, explaining both the reasons for and the concerns about certain interventions. This communication often facilitates a harmonious understanding between me and the client. Interestingly, yesterday another midwife told me there was a client who didn't want fetal monitoring or other interventions during labor ... I personally admitted to feeling anxious in such situations, given my tendency to adhere to established protocols and maintain a sense of control in my practice.’* (P13)

The possibility of facing formal complaints from clients about one’s performance or having to appear before a disciplinary board also caused feelings of anxiety. Moreover, some participants were aware of the relationship between their personal characteristics and their anxiety. Because they were so motivated by caring for others, satisfying their clients and, above all, doing what was right for the pregnant women, they developed feelings of anxiety about not being able to achieve this:

*‘Yes, that you don't do something as you should, or the possibility of failing to convey information to a client in its intended manner, ultimately resulting in client dissatisfaction. I think every midwife will always be afraid of that …’* (P2)

*The work environment*

Some participants mentioned that when their medical supplies were well organized and they knew where to find them, this gave them peace of mind. Furthermore, a familiar work environment reduces midwives’ anxiety. Moreover, participants experienced that the physical hospital environment reduced their feelings of anxiety.

*‘ … this applies most during the birth, where it is imperative to meticulously coordinate all relevant aspects. That you prepare well, to the extent possible, ensuring comprehensive organization. That you make sure you have everything [all needed materials] sorted out and that you, um, yes, how do you say that. Thereby fostering a sense of effective management over all controllable variables.’* (P16)

When pregnant women are referred to hospital, in some cases the community midwife remains involved in the woman’s care, providing midwifery care in the hospital environment for the duration of the birth. The proximity of colleagues who are available to help reduces their feelings of anxiety. Some participants experienced that working alone in the community without colleagues immediately available increased their anxiety:

*‘ … If someone wants to give birth at home, and I think: “you must be joking, I'm not going to do that!” Then I just go outpatient [transfer to the hospital because there are other obstetric colleagues to help if necessary]. That way, I can reduce the fear.’* (P1)

Resources in the work environment, such as collaboration with midwifery colleagues and colleagues from the multidisciplinary team, were perceived both as helpful and as a hindrance in reducing anxiety. On the one hand, community midwives found that having a supportive team in a safe environment reduced their feelings of anxiety. This included being able to talk freely about thoughts, feelings and situations:

*‘… and because once I had a CPR where an ambulance was there. And then I was very happy that they were there. I heard bad heart sounds, and I thought I'm just going to call them, I'll see what happens. And then the child came out, and he was doing really badly. And in the end, I did the CPR on my own, but they were there for support ... I just knew if I can't manage, they'll take over, so that gave a lot of support.’* (P9)

On the other hand, the work environment could also lead to increased feelings of anxiety. Insufficient support, tensions between the multidisciplinary team members, and a lack of confidence in their own professional competence increased participants’ anxiety. Participants perceived a lack of support from colleagues, in discussions about their perceptions of fear and anxiety. In their perception, this lack of peer support regarding work-related anxiety was caused by a taboo:

*‘ ... I really think that in our work, it is hardly mentioned, that it is often very much like, oh well, we all just do that. That doesn't bother us at all. While in the meantime, it's just bullshit because if you talk a bit more with colleagues one on one, then there's always that atmosphere of you don't complain. You just work. If you have a negative experience, that is part of your job, and we all just do that … So really a bit of the medical mentality that we have as doctors and midwives.’* (P16)

### Implications for performance

*Defensive behavior*

Participants who were aware of their feelings of fear and anxiety described it as a dilemma whether they should act on this anxiety. They mentioned that this dilemma influenced the process of clinical decision-making. Participants who described that they listened to the feelings of anxiety indicated that this sometimes affected their clinical decision-making. Other participants described that the feelings of anxiety only led to more conscious behavior without direct consequences:

*‘I think, during childbirth, acknowledging or exploring your fear, of: “Is it real? Or is it just mine?”. This also allows you to differentiate, “Should I take action now or should I just sit quietly and do something else for a while...?”.’* (P18)

Participants who were aware of the feeling of anxiety described that this could positively influence their professional behavior. This feeling could lead to consulting a colleague, accurate documenting or acting strictly according to the protocols, and deploying an intervention earlier. Interventions could vary from prevention (e.g. catheterizing, actively giving oxytocin after birth) to referring to hospital.

Participants mentioned that the feeling of anxiety increased after a negative experience, such as an obstetric emergency or negative feedback from a colleague. Participants described that most of the time, anxiety faded away after some time:

‘And obviously, I also have those periods, and then you really think for a moment, then you’re at a home birth again, and then, then I'm really a bit more nervous, then I'm really a bit more on top of it, and then, um, maybe intervene a bit faster and stuff, and that has to reduce, that will come back automatically when you have experienced more often that things are going well and I also see it with colleagues, so that is absolutely true.’ (P6)

*Offensive behavior*

Some participants indicated that they did act on confidence instead of listening to their feelings of anxiety and fear. They stated that they were confident in their own decisions and that, if a personalized policy was required, they would adapt a standardized protocol to individual needs after careful consideration and consultation with colleagues in their own team and in the hospital. Good contact with the client, confidence in their personal knowledge, and work experience that made them more aware of their actions enhanced these feelings:

*‘I had that nasty delivery on Wednesday. And Saturday night, I had another delivery. And then, of course, it played in my head, like yes, the same setting. But I put that aside really quickly, and uh ... No, um, yes, just a new situation and knowing that third babies are just sneezed out. With that thought, um, that's something I experienced that Wednesday is very unique because a third child with shoulder dystocia, well, come on. Who gets that, you know. So I've made that very clear to myself. And just thought when I stepped in there, of course, with some doubt, yes, no, let me put it this way, it's a new situation, and that's how I've really tried to try and handle it. And, um, just thought it's a third child, I just follow, or she should just follow her body.’* (P5)

*Decreased wellbeing*

Midwives mentioned that feelings of anxiety resulted in a decrease in their own wellbeing:

*‘I find the job quite mentally taxing at times. Sometimes I think, I could just work as a cash register. Then nobody would die. The worst thing that could happen would be a cash discrepancy at the end of the working day.’* (P15)

In addition, they mentioned the potential longer term consequences of anxiety on their career, ranging from reduced enjoyment in their work to attrition from the profession. Working with anxiety and fear also affected midwives’ job satisfaction. For some participants, defensive behavior and working strictly according to protocols were less satisfying. They choose to work with and have confidence in the physiological processes of pregnancy and childbirth:

*‘I have to think hard, yes. Well, if I don't know how to take that in hand, then for me personally, well, the consequence is that it will be less and less fun, I think. Because then I always react out of fear and dare or trust fewer things.’* (P7)

## DISCUSSION

In this study, we explored the perceptions of Dutch community midwives about fear and anxiety in practice and its impact on their performance. Our findings show that midwives were not used to talking about their work-related fear and anxiety. The interviews revealed four themes related to fear and anxiety: 1) perceived fear and anxiety, 2) the influence of years of experience, 3) the influence of the work content; and 4) the implications for performance. They experienced regular levels of fear in acute situations, such as obstetric emergencies where a pregnant client or a baby was in danger. Colleagues and clients could be perceived as increasing or reducing anxiety. Perceptions of anxiety were dependent on the amount of work experience as a midwife and the availability of colleagues and resources in the work environment. Participants expressed that fear and anxiety affected their performance in practice, whereby their wellbeing and job satisfaction decreased through a risk-avoidance attitude. Risk avoidance was expressed in the participants’ performance by more extensive reporting and low-threshold intervening.

The perceptions of Dutch midwives are similar to the outcomes of studies of Dahlen et al.^7,24^. They distinguished real fear and manufactured fear, which in this study were defined as fear and anxiety. Dahlen et al.^7,24^ recommend midwives to work with awareness of their feelings while maintaining their performance. For midwives in this study, talking about fear and anxiety might be the first step to becoming aware of their fear and not be guided by it. Although it is known that collaboration and peer support help to reduce fear^7,19^, for Dutch community midwives in this study, it did not seem to be common practice. In our study, midwives mentioned that talking to colleagues or relatives about negative experiences and traumatic events could be helpful and important. However, our results suggest that these conversations do not seem to happen very often in practice. The participants’ stories revealed different perspectives on fear and anxiety in midwifery. They recognized fear or anxiety in their colleagues, but not all midwives recognize their own fear as an obvious part of being a midwife. The importance of talking about fears and understanding fear as a part of the midwifery profession is internationally recognized^24,25^. Additionally, the conversations contribute to fear and anxiety as part of the work as a midwife, and these discussions are valued and recognized by fellow midwives^7^.

Fear, as described in this study, shows similarities with the causes of fear in previous research^7,9,11^. Fear of perinatal mortality, fear of missing information and causing harm, fear of blame for incidents and legal ramifications, fear of obstetric emergencies, fear of previous negative experiences and fear of recurrence, have all been reported in international studies^7,24^. Our findings show that midwives deal with anxiety all the time and it seems to be an obvious ‘part of the job’. However, similar situations may be distressing for one midwife and empowering for another. Midwives are known to differ in how they cope with adverse situations and stress^26^. One review found that some midwives managed stress by seeking support, journaling and self-reflection. Other midwives seemed to spiral downwards, experiencing flashbacks and nightmares, and subsequently reconsidering their midwifery career^26^.

Similar to previous research, our findings suggest that having years of practical work experience (i.e. managing acute situations) may in some way help to reduce fear and anxiety^14^. On the other hand, our findings show that previously acquired experiences could also increase midwives’ anxiety. The relationship between fear experienced in acute situations and midwives’ performances afterwards in this study could either lead to a constructive or a destructive effect on midwives. These two different attitudes were also mentioned in the findings of Offerhaus et al.^3^. Some midwives ‘emphasize physiology’, while other midwives are ‘operating on the safe side’, leading to more risk-avoiding behavior and performing according to protocols. A previous study by Healey et al.^9^ suggests that midwives show greater risk awareness and stricter clinical decision-making after a negative or traumatic event. This effect on clinical decision-making and risk awareness did not seem to apply only to that specific situation but also appeared to have an effect on future performances^25,27^. Our findings, however, revealed another mechanism: midwives whose awareness led to them opposing risk-avoidant behavior, and who are even more motivated for physiological care. They make a shift in their risk awareness and perform according to their own experienced-based decision-making. However, in these situations, they have to justify why they deviate from the protocol and on the basis of which arguments.

Our study demonstrated that the perceived anxiety can be caused by working soloistic in the community. This finding differs from international studies exploring anxiety, in which midwives are mostly hospital-based. The lack of an available colleague nearby when working in the community for home births affected community midwives: they felt more vulnerable and reported anxiety^14^.

Our findings show that colleagues can have both a positive and a negative impact on midwives’ anxiety. On the one hand, communication and collaboration with nearby colleagues and other team members in maternity care, and with clients, could reduce fear and anxiety^3,13,14^. On the other hand, a lack of mutual trust between midwives and colleagues, or midwives and clients, seems to reinforce midwives’ anxiety. This is similar to the findings of other Dutch studies in which increased levels of anxiety were found among community midwives without peer support compared to hospital-based midwives with available colleagues in their teams^13,14^.

### Strength and limitations

A strength of this study was our sample, which shows a variation of midwives’ backgrounds, with differences in years of work experience, age, and type of practice. We also achieved data saturation in the interviews and did a member check on the transcripts. A limitation of this study is that the interviews were carried out by six different pairs of students. Midwives may be less open with students about their experiences due to differences in experience levels.

### Implications

This study highlighted the importance of discussing work-related fear and anxiety in community midwifery practice. Talking about fear and anxiety helps to reduce these feelings^7,24^. The importance of acknowledging fear and anxiety as a professional issue has to be addressed by colleagues working in the community. The impact of fear and anxiety on midwives’ performance and wellbeing has to be addressed and recognized within the profession. Although fear and anxiety can lead to increased attention in some situations, it is a normal part of human behavior. However, following a traumatic birth, anxiety can also lead to a stress response, which in the long-term can reduce well-being^28^. Organizing adequate support for midwives after traumatic events and follow-up support for midwives on a regular basis will help to build a resilient profession^24^. This is even more the case for community midwives who work alone. Midwives need to rebuild their confidence in physiological birth after adverse events. To rebuild their confidence, they need a temporary support system within the practice organization. Furthermore, the importance of supporting relationships between team members in monodisciplinary and multidisciplinary teams as a tool for reducing fear and preventing anxious performance in practice must be addressed.

Moreover, there must be awareness of the impact of clients’ high expectations of midwives. This could affect feelings of anxiety and their performance. Midwives have to recognize the importance of trust in the relationship between client and care provider. They also have to learn to communicate their intuitive feelings when they feel a lack of trust in the relationship.

Further research is recommended to quantify the results on work-related fear and anxiety among midwives in community settings. A recommendation for education is to focus more on the professional development of student midwives in coping with fear and anxiety.

## CONCLUSIONS

Midwives expressed similar feelings of fear as recognized in previous studies. As well as feelings of fear, anxiety seemed to exist in community midwives. Experiencing more adverse situations and societal challenges towards risk aversion in pregnancy and childhood seemed to affect midwives. With more years of experience, feelings of anxiety can both increase and decrease, which seemed to depend on midwives’ awareness of their own fears and anxieties.

## Data Availability

The data supporting this research cannot be made available for privacy or other reasons. We cannot guarantee the privacy of the midwives who participated in this study.
